# Bruxism, Related Factors and Oral Health-Related Quality of Life Among Vietnamese Medical Students

**DOI:** 10.3390/ijerph17207408

**Published:** 2020-10-12

**Authors:** Nguyen Thi Thu Phuong, Vo Truong Nhu Ngoc, Le My Linh, Nguyen Minh Duc, Nguyen Thu Tra, Le Quynh Anh

**Affiliations:** 1 School of Odonto Stomatology, Hanoi Medical University, Hanoi 100000, Vietnam; drnguyenthuphuong70@gmail.comit (N.T.T.P.); nhungoc@hmu.edu.vn (V.T.N.N.); lemylinh.hmu@gmail.com (L.M.L.); qule7436@uni.sydney.edu.au (L.Q.A.); 2 Division of Research and Treatment for Oral Maxillofacial Congenital Anomalies, Aichi Gakuin University, 2-11 Suemori-dori, Chikusa, Nagoya, Aichi 464-8651, Japan; 3 School of Dentistry, Faculty of Medicine and Health, The University of Sydney, Sydney, NSW 2000, Australia

**Keywords:** bruxism, oral health-related quality of life, perceived stress, educational stress, Vietnamese medical students

## Abstract

Although bruxism is a common issue with a high prevalence, there has been a lack of epidemiological data about bruxism in Vietnam. This cross-sectional study aimed to determine the prevalence and associated factors of bruxism and its impact on oral health-related quality of life among Vietnamese medical students. Bruxism was assessed by the Bruxism Assessment Questionnaire. Temporomandibular disorders were clinically examined followed by the Diagnostic Criteria for Temporomandibular Disorders Axis I. Perceived stress, educational stress, and oral health-related quality of life were assessed using the Vietnamese version of Perceived Stress Scale 10, the Vietnamese version of the Educational Stress Scale for Adolescents, and the Vietnamese version of the 14-item Oral Health Impact Profile, respectively. The prevalence of bruxism, sleep bruxism, awake bruxism, and both conditions in Vietnamese medical students were 51.2%, 38.2%, 23.4%, and 10.4% respectively. Stress, temporomandibular joint pain, masticatory muscle pain, and tooth attrition were associated with the presence of bruxism. Vietnamese medical students were negatively affected by bruxism in terms of oral health-related quality of life.

## 1. Introduction

Bruxism is defined as “a repetitive jaw-muscle activity characterized by clenching or grinding of the teeth and/or by bracing or thrusting of the mandible”. Bruxism has two manifestations based on occurring time: sleep bruxism (SB) occurring during sleep and awake bruxism (AB) occurring during wakefulness [1]. A systematic review in adult subjects [2] described an 8 to 31% prevalence of generical bruxism. In detail, the prevalence of sleep bruxism was from 12.8 ± 3.1%, and awake bruxism was from 22.1 to 31%.

There are many etiological factors and associated factors of bruxism: peripheral factors such as teeth occlusion interferences, central or pathophysiological factors, and psychological factors such as stress [3]. Previous studies have reported the relation between bruxism and temporomandibular joint (TMJ) pain, TMJ noise, limited mouth opening, abnormal opening pathway, articular disc dislocation, and osteoarthritis [4,5,6]. Rosales et al. using animal models showed that inducing emotional stress caused brux-like activities in the masseter of rats [7]. A number of studies concluded that individuals with bruxism tend to report more symptoms of stress and anxiety than non-bruxism people [8,9].

Oral health plays a crucial role in an individual’s general health. An oral problem can lead to changes in oral manifestations, consequently affecting all aspects of life physically and psychologically. Previous studies showed bruxers had a worse oral health-related quality of life (OHRQoL) than normal people. These studies described significantly higher OHIP-14 scores of bruxers than controls.

Although bruxism is a common issue with a high prevalence reported in previous studies, there is a significant lack of epidemiological data about bruxism in Vietnam. To our best knowledge, there have been no bruxism related studies conducted in Vietnamese people or Vietnamese students. Thus, we conducted this study to investigate: (i) the prevalence of bruxism, (ii) associations between bruxism and stress, temporomandibular disorder (TMD), and tooth attrition, and (iii) effects of bruxism on the OHRQoL among Vietnamese medical students.

## 2. Materials and Methods

### 2.1. Subjects

From Hanoi Medical University (Hanoi, Vietnam) 746 students were recruited for the current study. One-hundred-and-twenty participants who regularly took neurological system-related drugs (anti-epileptics, anxiolytic), or wore removable dental prostheses (partial or complete removable dentures, orthodontic appliances), or received any bruxism treatments in the last six months were excluded. From these, 626 students were taken dental history and received an oral examination by experimented dentists. Individuals with active dental caries, endodontic diseases (pulpitis), periodontitis, or reporting these conditions in the last six months were also excluded. Finally, there were 568 students (305 females, 263 males, mean age: 22.2 ± 0.4) involved.

Ethical approval (rhm-2019-181) was issued by the Professional Council of School of Dentistry, Hanoi Medical University.

### 2.2. Bruxism Diagnosis

Bruxism diagnosis is graded as possible, probable, and definite bruxism. Self-reported bruxism was considered as an indication of possible SB or AB [1]. The current study exclusively used self-reports in bruxism diagnosis, thus it could only identify ‘possible bruxers’. Possible SB was evaluated by a questionnaire recommended by the American Academy of Sleep Medicine [10]. Possible AB was detected by two questions developed by Pintado et al. [11,12]:

1. Are you aware, or has anyone heard you, grinding your teeth frequently during sleep? (yes/no)
2. Are you aware that your dentition is worn down more than it should be? (yes/no)
3. Are you aware of any of the following symptoms upon awakening? (yes/no):
  (i) Sensation of fatigue, tightness, or soreness of your jaw upon awakening?
  (ii) Feeling that your teeth are clenched or that your mouth is sore upon awakening?
  (iii) Aching of your temples upon awakening?
  (iv) Difficulty in opening your mouth wide upon awakening?
  (v) Feeling of tension in your jaw joint upon awakening and feeling as if you have to move your lower jaw to release it?
  (vi) Hearing or feeling a ‘‘click’’ in your jaw joint upon awakening that disappears afterward?
4. Are you ever aware of grinding your teeth during the day? (yes/no)
5. Are you ever aware of clenching your teeth during the day? (yes/no)

Respondents were determined as suffering from possible SB if their answer was “yes” to question 1 and⁄or question 2 or at least one “yes” answer to a symptom listed in question 3. The answer of “yes” to Question 4 and/or question 5 indicated that the participant had possible AB.

### 2.3. Temporomandibular Disorders

TMD was diagnosed by a single experienced dentist through clinical examination and history interviews following the Diagnostic Criteria for Temporomandibular Disorder 2014 (DC/TMD 2014) Axis I [13]. Masticatory muscle pain was defined as the pain in masticatory muscles. The pain was confirmed as the familiar pain in relevant masticatory muscles induced by palpation. Temporomandibular joint (TMJ) pain was defined as the pain in the front of the ear induced by mouth opening and chewing or TMJ palpation. TMJ sounds were defined as clicks, popping, or crepitus occurring in opening and closing mouth. The abnormal mouth opening pathway was defined as the deflection or deviation of the mouth opening pathway. Limited mouth opening was defined as the maximum range of mouth opening narrower than 40 mm.

### 2.4. Perceived Stress

The level of emotional stress was measured by using the Vietnamese version of the Perceived Stress Scale 10 (PSS-VN10) [14]. The questionnaire consisting of 10 items examines stressful feelings and thoughts from which respondents had suffered during the past month. Respondents were asked to rate the frequency he ⁄she felt ⁄thought on a scale of four ranging from ‘‘never’’ to ‘‘very often’’, with a total score ranging between 0 and 40 (a higher score indicating a higher level of emotional stress). A high level of perceived stress was defined as a score ≥20.

### 2.5. Educational Stress

Educational stress was measured by the Vietnamese version of the Educational Stress Scale for Adolescents (ESSA-VN) [15]. Responses to the 16 items are on a 5-point Likert-type scale ranging from 1 (strongly disagree) to 5 (strongly agree). We adjusted the questionnaire to adapt to student subjects by eliminating questions number 5 and 10. Thus, the total score of this part ranged from 14 to 70. A higher score indicated a higher level of emotional stress. A high level of educational stress was defined as a score ≥42.

### 2.6. Tooth Attrition

Participants were clinically examined for assessing visual tooth attrition. Tooth attrition was detected if there was incisal wearing in incisors, canines, or wearing in occlusal surfaces of premolars, molars.

### 2.7. Quality of Life

The OHRQoL was evaluated by the Vietnamese version of the OHIP-14 questionnaire [16]. The responses of each question were a 5-levels scale: 0—never; 1—almost never; 2—sometimes; 3—fairly often; 4—very often. The OHIP-14 score is the sum scores of 14 responses, ranges from 0 to 56, the score of each issue ranges from 0 to 8. The total score of the OHIP-14 provides a comprehensive view of the effects of oral health on the quality of life.

### 2.8. Statistical Analyses

All the collected data were analyzed using SPSS for Windows (IBM, Armonk, NY, USA) version 20.0. Relations between bruxism and perceived stress, educational stress were tested by a Binary Logistic regression model. Relations between bruxism and TMD (TMJ pain, TMJ noise, Opening pathway, Limited mouth opening, Masticatory muscle pain), tooth attrition were tested by Chi-square. Once the relations were detected, a further investigation using multiple logistic regression would be done for the related factors to finally confirm. Quality of life and the presence of bruxism were analyzed using an independent sample t-test. The significance level was set at 0.05 for all tests.

## 3. Results

### 3.1. Prevalence

There were 291 students (51.2%) diagnosed with possible bruxism. No significant difference was observed between females and males (*p* = 0.425) (Table 1). The prevalence of SB and AB were 38.2% and 23.4%, respectively. The prevalence of those suffering from both sleep and awake bruxism was 10.4%.

### 3.2. Associated Factors

Binary Logistic regression models showed that perceived stress and educational stress had statistically significant correlations with the presence of bruxism (*p* < 0.001) (Table 2).

Chi-square analyses showed that the presence of bruxism significantly related to TMJ pain, TMJ noise, masticatory muscle pain, abnormal opening pathway, and tooth attrition (*p* < 0.001) (Table 3).

In the model of multiple logistic regression, there were four factors: perceived stress, TMJ pain, masticatory muscle pain, and tooth attrition. These remained the association with the presence of bruxism (Table 4).

### 3.3. Effects on Quality of Life

Independent sample *t*-test comparing the life quality indices between bruxism and non-bruxism groups showed that subjects who suffered from bruxism had significantly higher OHIP-14 scores in all issues and the total score (Table 5).

## 4. Discussion

The prevalence of bruxism among Vietnamese medical students (51.2%) was higher than the results reported in previous studies, from 8–31% [2]. However, the indirect comparisons from the literature should be interpreted with caution because diagnosis methods, clinical criteria, and samples of the population varied between studies, for example, convenient samples, restricted geographical area samples, or samples selected in workplaces. The current study was conducted on medical students. Students suffer a stressful study process during their undergraduate course which causes high levels of stress to be reported in students [17]. Other sources of stress could derive from time or economic problems [18]. Medical students have higher levels of stress than their same-age peers [19,20,21]. Abdulghani et al. reported that 63% of medical students experience stress, half of it being severe [21]. A positive correlation between bruxism and stress was reported among students [22]. Different studies also revealed relative high prevalences of bruxism, 31.6% [23], 36.5% [24], 37.9% [22], in student subjects. Therefore, a high prevalence of bruxism in this study was expected. Moreover, a study comparing the prevalence of bruxism detected by interview and polysomnography showed that the result done by the interview method (12.9%) was higher than by polysomnography (7.4%) [25]. Our study used only self-report to diagnose ‘possible bruxers’, which could overestimate the real prevalence.

In the current study, SB (38.2%) was observed more commonly than AB (23.4%). This result was similar to the results of previous studies [26,27], while others showed a reverse trend [28,29,30]. Even though the interview is the most common method used in community studies due to its simplicity, this method is only efficient to diagnose participants with symptomatic bruxism or awareness of their bruxism. Eighty percent of SB cases are noiseless and unconscious [11]. Moreover, the difference between AB and SB is hard to be found during clinical examination since both types have numerous similar signs and symptoms. Hence, SB might be easily omitted or misreported.

Multiple logistic regression models revealed that the presence of bruxism was associated with perceived stress, which was also reported in previous studies [31,32]. Literature showed that people in every age suffering from stress are more likely to have bruxism [33,34,35]. A study of Winocur et al. [36] on Israel young adults using the PSS scale showed the probability of bruxism increased 3.2–3.3 folds in subjects with a higher level of the PSS scale. Several investigations on animal models proved bruxism could be occurred by evoking psychophysiological mechanisms [7,37,38]. Chronic stress, caused by physical or emotional stimuli, can activate the dopaminergic system, which may cause non-functional masticatory movements.

Masticatory muscle pain, tooth attrition, and TMJ pain were also observed in associations with bruxism. Both clenching and grinding increase the activity of masticatory muscle leading to overwork of muscle, vasoconstriction, accumulation of intermediate metabolites, and resulting in ischemia. Inside the ischemic area, noxious chemicals as bradykinin, prostaglandin, and protons are released, causing pain to muscle. A study using polysomnography suggested that bruxism is related to hyperactivity of masticatory muscle and the increase in neuromuscular signals in masticatory muscles [4,5]. Tooth attrition is caused by tooth-tooth contact, leading to wore out of the occlusal surface and incisor edge. Physiological activities also lead to attrition, such as chewing and swallowing. However, in patients with bruxism, attrition becomes worse due to the increase in the duration of tooth-tooth contacts [39]. Bruxism can increase the TMJ load, resulting in signs and symptoms of TMD [40]. Vivo studies showed that acute mechanical overloads can cause severe disc damage to the TMJ [40,41].

The relation between bruxism, TMJ noise, limited mouth opening, and abnormal opening pathways was not found in this study. This can be explained by the signs and symptoms of TMD being related to many issues. For instance, an abnormal opening pathway is usually associated with joint pain and muscle pain [42]. Crepitus is one of the signs of osteoarthritis which is a degenerative joint disease including pain [43]. In the multiple logistic regression model, the role of TMJ noise and abnormal mouth opening might be dominated by other TMD symptoms, such as masticatory muscle pain and TMJ pain. Chi-square analysis revealed no relation between bruxism and limited mouth opening, possibly, because the number of subjects that had limited mouth opening was only seven.

In terms of the OHIP-14, students with bruxism had higher scores in the total score and all domain scores of the OHIP-14 than students without bruxism, suggesting a poorer OHRQoL in bruxers compared to non-bruxers. The physical pain domain (painful aching, uncomfortable to eat) obtained the highest average score, which is similar to previous studies [44,45]. Most bruxism associations found in the current study are related to the pain sensation: masticatory muscle pain, TMJ pain, and tooth attrition which may accompany tooth hypersensitivity. Pain both physically and mentally affects health, and usually is the first thing to be acknowledged [46]. Hence, bruxism, via pain, might have negative impacts on the OHRQoL in bruxism subjects. Regarding the physical disability (unsatisfied diet), functional limitation, and handicap domains, the higher scores in students with bruxism were also found. Tooth attrition condition in bruxers which requires more chewing cycles [47], impedes breaking hard and chewy foods, and causes tooth hypersensitivity (triggered by cold, hot, or sour foods) subsequently affects diets [48,49]. As aforementioned, pain is associated with physical and mental health, thus the effects of pain in bruxism patients include all psychological and physical problems. These above issues can cause difficulties in concentrating, interference with daily works and activities, and affect social communications. Psychological problems are more common in bruxism patients than the general population [33,34,36]. Previous studies revealed that bruxism-related traits include aggressiveness, hypersensitivity to stress, anxiety, depression, and fearfulness [34,44]

Our study investigated bruxism based on self-report only. Therefore, there was a limitation in obtaining a definite prevalence, only the presence of ‘possible bruxism’ could be detected, which might lead to a significantly higher prevalence reported than reality. Further studies need to improve diagnosis criteria by combining self-report with clinical examination and polysomnography to increase the accuracy of results.

## 5. Conclusions

The prevalence of bruxism among Vietnamese medical students was 51.2%. Four factors including perceived stress, TMJ pain, masticatory muscle pain, and tooth attrition were associated with the presence of bruxism. Bruxism negatively affects the life quality of Vietnamese medical students.

## Figures and Tables

**Table 1:** The prevalence of bruxism among Vietnamese medical students.

Index	Female	Male	Total	p
Prevalence	52.8%	49.4%	51.2%	0.425

**Table 2:** Relations between the presence of bruxism and perceived stress, educational stress.

Factors	Odd Ratio (OR)	95% Confident Interval (CI)	*p*
Perceived Stress	3.02	2.00–4.57	<0.001
Educational stress	2.05	1.42–2.95	<0.001

**Table 3:** Relations between the presence of bruxism and associated factors.

Factors	Answer	Bruxism *n* (%)	No Bruxism *n* (%)	OR (95% CI)	*p*
TMJ pain	Yes	78 (84.8)	14 (15.2)	6.88 (3.78–12.50)	<0.001
	No	213 (44.8)	263 (55.2)		
TMJ noise	Yes	112 (65.5)	59 (34.5)	2.31 (1.59–3.35)	<0.001
	No	179 (45.1)	218 (54.9)		
Opening pathway	Normal	119 (63.3)	69 (36.7)	2.09 (1.46–2.98)	<0.001
	Abnormal	172 (45.3)	208 (54.7)		
Limited mouth opening	Yes	3 (42.9)	4 (57.1)	0.71 (0.16–3.21)	0.719
	No	288 (51.3)	273 (48.7)		
Masticatory muscle pain	Yes	187 (91.7)	17 (8.3)	27.5 (15.93–47.48)	<0.001
	No	104 (28.6)	260 (71.4)		
Tooth attrition	Yes	169 (81.6)	38 (18.4)	8.71 (5.76–13.18)	<0.001
	No	122 (33.8)	239 (66.2)		

TMJ—tempomandibular joint.

**Table 4:** Multiple logistic regression model of the relation between perceived stress, educational stress, temporomandibular joint (TMJ) pain, TMJ noise, masticatory muscle pain, abnormal opening pathway, and attrition the presence of bruxism.

Factors	OR	95% CI	*p*
Perceived stress	2.86	1.57–5.19	0.001
Educational pressure	0.98	0.56–1.71	0.995
TMJ pain	2.76	1.23–6.19	0.014
TMJ noise	0.83	0.34–1.99	0.667
Opening pathway	1.22	0.52–2.90	0.564
Masticatory muscle pain	34.63	18.08–66.33	<0.001
Tooth attrition	14.25	8.19–24.80	<0.001

**Table 5:** Effects of bruxism on the oral health-related quality of life.

Issues/Problems	Bruxism Mean Score ± SD	Non-Bruxism Mean Score ± SD	Mean Difference	95% CI	*p*
Functional limitation	1.4 ± 1.4	0.8 ± 1.1	0.65	0.44–0.86	<0.001
Physical pain	3.3 ± 1.4	2.6 ± 1.6	0.78	0.53–1.03	<0.001
Psychological discomfort	3.0 ± 1.7	2.1 ± 1.7	0.91	0.63–1.20	<0.001
Physical disability	2.0 ± 1.6	1.3 ± 1.5	0.69	0.44–0.94	<0.001
Psychological disability	2.4 ± 1.7	1.5 ± 1.5	0.88	0.62–1.15	<0.001
Social disability	1.4 ± 1.5	0.7 ± 1.2	0.65	0.42–0.87	<0.001
Handicap	1.4 ± 1.5	0.8 ± 1.2	0.64	0.42–0.87	<0.001
Total score	14.9 ± 8.0	9.7 ± 7.9	5.19	3.08–6.50	<0.001
